# Health, risk behaviour and consumption of addictive substances among physicians - results of an online survey

**DOI:** 10.1186/s12995-018-0208-7

**Published:** 2018-08-23

**Authors:** Dominik Pförringer, Regina Mayer, Christa Meisinger, Dennis Freuer, Florian Eyer

**Affiliations:** 1 Department of Trauma Surgery, Technical University of Munich, Ismaningerstrasse 22-, 81675 Munich, Germany; 2 Chair for Epidemiology at UNIKA-T, Ludwig-Maximilians University of Munich, Augsburg, Germany; 3 Klinikum rechts der Isar, Department of Clinical Toxicology, Technical University of Munich, Munich, Germany

**Keywords:** Health, Physicians’ health, Addiction, Risk behaviour, Alcohol, Drugs, Consumption, Online survey

## Abstract

**Background:**

Previous studies were able to show that hazardous alcohol and substance abuse among physicians is not rare. Currently no recent data to detect risk groups are available either on the prevalence of hazardous drinking disorders and risky health behaviour among physicians or on influencing factors (age, gender, role, institution, specialization, working hours).

**Methods:**

A 42-item online questionnaire was distributed to 38 university hospitals, 296 teaching hospitals and 1290 physicians in private practice. The questionnaire addressed health behaviour and alcohol/substance consumption as well as demographic and work-related properties.

**Results:**

Out of 1338 a total of 920 questionnaires could be evaluated. 90% of physicians estimate their health status as satisfying. 23% of doctors consume hazard quantities of ethanol, 5% are nicotine addicted, and 8% suffer from obesity. Childlessness (*p* = 0,004; OR = 1,67; KI = 1,17-2,37) for both genders and the role of a resident for females (*p* = 0,046, OR = 3,10, KI = 1,02-9,40) poses a risk factor for hazardous alcohol consumption. Weekly working hours of more than 50 h (*p* = 0,009; OR = 1,56; KI = 1,12-2,18) and a surgical profession (*p* < 0,001; OR = 2,03; KI = 1,47-2,81) may also be a risk factor towards hazardous and risky health behaviour.

**Conclusion:**

A more structured and frequently repeated education on help offerings and specific institutions for addicted and risk groups seems essential.

## Background

Aspects of health attitude and consumption of addictive substances among doctors are frequently found in media and science [1–6]. Not only overburdening workload and burnout but alcohol and medication abuse also play a significant role. One major problem is the taboo status of discussing such behaviour in a group of co-workers exposed to high pressure and stress. Geuenich showed in 2009, that 46% of doctors in Germany suffer from psychological pressure both in the professional as well as the social context [7]. Information on available help and support as well as solutions are not commonly well known, even though they exist [4, 8–10]. In the US, lifetime prevalence of alcohol abuse and dependence of doctors in 1992 was estimated at 6%, while in Norway hazardous alcohol consumption among doctors increased from 12 to 15% between 1993 and 2000 [11, 12].

However, little scientific information on German doctors exists – prevalence of hazardous alcohol consumption according to Rosta et al. 2008 was 20% [1, 13, 14]. In a survey study, Unrath et al. determined in 2012 that almost every fourth general practitioner in Rhineland-Palatinate consumes alcohol on a daily basis [14]. Burnout and depression among physicians as well as addiction and risky health behaviours are under-represented clinical pictures [15, 16]. Mäulen assumes that alcohol is the most common drug used by doctors [10, 17]. In a survey study in 2008 Rosta found that particularly surgical disciplines and male doctors pose risk groups for hazardous alcohol consumption, but age has no significant influence [13]. However, other influencing factors were not investigated, and the influence of age, sex and discipline was not analysed in a multivariable way. In a smaller study in 2009, Voigt et al. studied the smoking behaviour of physicians in Saxony and Brandenburg - 86% were non-smokers [18]. Furthermore, experts estimate that about 0.7% of physicians in Germany use narcotic drugs (BtM), but there is no reliable data regarding this [10]. There is currently no data on general risk-taking and the physical activity and nutrition of physicians in Germany.

Hazardous drinking is defined as the consumption of an amount of alcohol that increases the risk of harmful consequences for the user or others. Harmful drinking on the other hand comes along with actual physical or mental harm [19]. Since the transition between these terms is fluent, we use the term ‚hazardous‘ for both terms [20]. The amount of alcohol which marks hazardous consumption is differently defined. For example, Reid et al. 1999 defined: “21 drinks or more per week for men (or >= 7 drinks per occasion at least 3 times a week) or 14 drinks or more per week for women (or >=5 drinks per occasion at least 3 times a week)” [21]. A more recent study of Wood et al. concluded that the consumption of more than 100 g alcohol per week is risky [22].

The aim of the present work is to update the data on addiction and health behaviour of physicians, as well as to reflect the general risk tolerance. Furthermore, risk groups can be identified to support the development of prevention strategies. The following questions were examined:

- How do doctors evaluate their own health?
- How balanced is a doctor’s diet and how regularly do they exercise?
- What is the general willingness of doctors to take risks and how often do they participate in preventive examinations? Do predisposed groups exist?
- What is the prevalence of dangerous alcohol use among physicians and how does it affect their counselling behaviour? Which groups are particularly at risk?
- What is the prevalence of substance use, risk taking and obesity in specific comparison between surgical and non-surgical specialties?

## Methods

### Design and conduction of survey

This study was conducted as an anonymous survey of physicians in Germany using web-based survey opportunities on the website of pollster SurveyMonkey® (San Mateo, CA) [16]. The survey was reviewed and approved by the Ethics Committee of the Faculty of Medicine of the Technical University of Munich (number: 555 / 15S).

In mid-October 2016, an e-mail cover letter was sent to medical and commercial directors and / or hospital management of the 38 university hospitals in Germany as well as to 296 teaching hospitals [23]. A maximum of nine teaching hospitals were contacted per university.

During the same period, 1290 physicians in 11 German federal states were contacted by e-mail after selection via a multi-stage random procedure, selected on the basis of the online registries of the medical associations. In each case, according to the area of the federal state, a representative sample stratified by discipline was drawn. A multiple of 15 general practitioners and one representative from 23 specialties was selected in total: Bavaria was chosen as a reference state, where a calculated total of 120 general practitioners and 184 members of other disciplines were examined.

The federal states were Bavaria (120/184), Baden-Wuerttemberg (80/115), Saarland (13/23), Rhineland-Palatinate (40/69), Hesse (40/69), Thuringia (40/69), Lower Saxony (90/138), Saxony-Anhalt (40/69), Bremen (6/12), Schleswig-Holstein (30/46) and Hamburg (7/11).

Areas of specialization were anaesthesiology, surgery, dermatology, gynaecology and obstetrics, internal medicine, child and adolescent psychiatry, laboratory medicine, nuclear medicine, pathology, plastic surgery, psychosomatics, urology, radiology, ear nose throat, human genetics, paediatrics, neurology, microbiology, orthopaedics and traumatology, physical and rehabilitative medicine, psychiatry and radiation therapy.

One out of ten in the online directories of the medical associations, sorted by subject, was selected. Since some federal states did not have enough representatives in the smaller subject areas, 21 physicians less were addressed - so a total of 1290 doctors instead of 1311.

To achieve greater participation and to maintain the anonymity of clinics and doctors, confirmation of participation in the survey by the respective clinic or respective physician was waived. The cover letter included a link to the survey (one-time redirection per IP address), a short description of the content and the request for internal and external distribution. The survey was open for participation for a total of 10 weeks (11th October to 19th December 2016). In mid-November 2016, a reminder letter was sent by e-mail to all contacted clinics and doctors. Surveys addressed to the relevant specialist societies in Austria, Switzerland and occasionally in English-speaking countries were not included in the evaluation due to insufficient numbers of participants.

### Questionnaire

By including validated tests and using literature, a 42-item questionnaire (41 plus consent) was developed using SurveyMonkey®'s online tool [24–32]. A team of eight physicians and four clinical researchers from various disciplines not involved in the study at Klinikum rechts der Isar tested the survey for comprehensibility as well as the quality and lack of questions. After evaluating the suggestions for improvement and the modification of six items, the final version was created. Answering the questions averaged about 7 min. The questionnaire can be found in the online supplement.

Included were questions on general health behaviour and attitude, sports, diet and socio-demographic as well as occupational aspects. To examine the nicotine and alcohol consumption behaviour validated tests were used. The former was examined by using the Heaviness of Smoking Index (HSI), a short form of the Fagerström Test for Nicotine Dependence (FTND) [33, 34]. The latter was examined by the AUDIT-C, a short form of the Alcohol Use Disorders Identification Test (AUDIT) with comparable psychometric quality [26, 32, 35]. A cut-off point of 5 or more was used to detect hazardous alcohol consumption – this sum was recommended as cut-off for the German population [36]. Additionally the CAGE questionnaire was integrated, whose answering was voluntary to avoid dropouts [30].

### Statistics

The evaluation was carried out by means of the statistical software SPSS (SPSS Statistics Premium 24, IBM). Depending on type of data, mean values ​​with standard deviations or relative and absolute frequencies were calculated for the descriptive analysis. Differences between physicians, surgical and non-surgical disciplines, as well as hazardous and non-hazardous alcohol consumption were analysed with Fisher’s exact test, chi-square test or the t-test for two unconnected samples, depending on the scale level. Furthermore, binary logistic regression analyses were performed in multivariate models, adjusted for potential influencing factors.

## Results

### Return and sample size

Altogether, in the German-language version of 1338 on-line questionnaires 1096 were completely filled out. Completion rate thus equals 81.9%. The response rate itself is not assessable in this study, because we refrained from retracing the participants to ensure anonymity and to achieve a greater number of participants. Twenty-two participants did not agree to the scientific use of their data and had to be excluded. Due to the low number of active doctors in Austria (*n* = 25) and Switzerland (*n* = 50), only 988 participants active in Germany were selected (excluded: other country: 4, missing information: 7). Forty-six people were excluded because they were not doctors or non-medics and another 22 because they gave missing or obviously wrong answers in the categories analysed here. Thus, the following results are based on the complete information of 920 physicians working in Germany.

The majority of participating physicians were younger than 35 years, whereas more men than women were over 46 years old (43 and 31%, respectively) (Table 1). Significantly more men than women lived with children in the household (52 and 38%, respectively), and they were also significantly more likely to be married (73 and 53%, respectively). Most participants came from internal medicine (21%), from surgery (including orthopaedics and trauma surgery) (18%) and anaesthesiology (16%). Nearly one in two men worked in a leadership position (45%), while just below half of the women were residents (46%). 85% of all responding physicians worked in a clinic. 53% of the female physicians and 71% of the male physicians regularly worked at least 50 h a week.

**Table 1:** Socio-demographic and occupational characteristics (absolute numbers/frequency in %)

Characteristic	Female physicians (*n* = 417)	Male physicians (*n* = 503)	All (*n* = 920)	*P*-value
Age group				
18–35	190/46	150/30	340/37	< 0,001
36–45	101/24	135/27	236/26	
46–55	90/22	131/26	221/24	
older than 55	36/9	87/17	123/13	
Marital status				
Unmarried	177/42	120/24	297/32	< 0,001
Married	221/53	365/73	586/64	
Divorced/widowed	19/5	18/4	37/4	
Children (living in the household)				
Yes	159/38	263/52	422/46	< 0,001
No	258/62	240/48	498/54	
Medical speciality				
General practice	32/8	45/9	77/8	< 0,001
Anaesthesiology	66/16	85/17	151/16	
Surgery^a^	28/7	52/10	80/9	
Orthopaedic and trauma surgery	16/4	66/13	82/9	
Internal medicine	87/21	107/21	194/21	
Paediatrics	38/9	21/4	59/6	
Psychiatry/child and adolescent	38/9	35/7	73/8	
Psychiatry				
Other	112/27	92/18	204/22	
Position/educational level				
Resident	193/46	145/29	338/37	< 0,001
Specialist	117/28	130/26	247/27	
Physician in a leading position	107/26	228/45	335/36	
Establishment				
University hospital	184/44	192/38	376/41	0.110
For-profit/public/non-profit hospital	177/42	224/45	401/44	
Phyiscian in private practice	56/13	87/17	143/16	
Working hours per week				
More than 70 h	24/6	58/12	82/9	< 0,001
60 h - less than 70 h	61/15	129/26	190/21	
50 h - less than 60 h	132/32	164/33	296/32	
40 h - less than 50 h	124/30	119/24	243/26	
30 h - less than 40 h	49/12	25/5	74/8	
Less than 30 h	27/7	8/2	35/4	
Stresses and strains				
Stress/pressure at work and in the private environment	157/38	173/34	330/36	0.590
Stress/pressure at work or in the private environment	210/50	266/53	476/52	
None	50/12	64/13	114/12	

^a^ without orthopaedic and trauma surgery

### Examined variables

There was no difference between the subjective perception of health by members of a surgical specialty and a non-surgical specialty. Health was found to be at least satisfactory by almost all (90%) (Table 2). Almost every second surgeon (44%) sometimes or often took risks, significantly more frequently than doctors in non-surgical disciplines (26%). Surgeons also took greater risks because of the fact that only one out of five regularly participated in check-ups. Also the AUDIT-C test suggested that at least 29% of surgeons had at least a hazardous alcohol intake - nearly one in four doctors in total. 8% of participating physicians were severely obese (BMI ≥30) and 5% showed moderate to high nicotine dependence in the Heaviness of Smoking Index.

**Table 2:** Health, risks, consumption of addictive substances in surgical and non-surgical specialties (absolute numbers/frequency in % or means [standard deviations])

Examined variables	Surgical speciality^a^ (*n* = 241)	Non-surgical speciality^a^ (*n* = 679)	All (*n* = 920)	*P*-Value
Health condition (subjective)				
Satisfactory/good/very good	217/90	610/90	827/90	1.000
Very poor/poor/less healthy	24/10	69/10	93/10	
Actively taking health risks				
Sometimes/often	107/44	175/26	282/31	< 0,001
Rarely/never	134/56	504/74	638/69	
Preventive check-ups				
Often	41/17	181/27	222/24	0.003
Rarely	200/83	498/73	698/76	
BMI				
	24,5 (3,8)	24,1 (3,5)	24,2 (3,6)	0.160
BMI - categories				
Short/normal weight (BMI < 25)	159/66	444/65	603/66	0.059
Overweight (BMI 25 bis < 30)	55/23	188/28	243/26	
Obesity (BMI ≥ 30)	27/11	47/7	74/8	
Nicotine^b^				
Moderate to high dependence	15/6	29/4	44/5	0.145
Low dependence	27/11	55/8	82/9	
Non-smoker/other^c^	199/83	595/88	794/86	
Alcohol^d^				
Hazardous alcohol consumption	70/29	142/21	212/23	0.013
Harmless/no alcohol consumption	171/71	537/79	708/77	
“How often do you drink alcohol in general?” (Scores)				
4 times or more often per week (4)	38/16	99/15	137/15	
2–3 times per week (3)	81/34	196/29	277/30	
2–4 times per month (2)	80/33	210/31	290/32	
Monthly or less (1)	26/11	111/16	216/24	
Never (0)	16/7	63/9		
“How many units^e^ of alcohol do you drink on a typical day when you are drinking?” (Scores)				
10 or more (4)	1/0	1/0	2/0	
7–9 (3)	6/3	9/1	15/2	
5–6 (2)	11/5	20/3	31/3	
3–4 (1)	59/25	113/17	172/19	
1–2 (0)	148/61	473/70	621/68	
I don’t drink alcohol at all. (0)	16/7	63/9	79/9	
“How often have you had 6 or more units of alcohol on one day in the last year?” (Scores)				
Daily or almost daily (4)	3/1	1/0	4/0	
Once a week (3)	12/5	21/3	33/4	
Once a month (2)	35/15	64/9	99/11	
Less than monthly (1)	93/39	200/30	293/32	
Never (0)	98/41	393/58	491/53	

^a^Surgical speciality: surgery, gynaecology, urology, ENT, ophthalmology; non-surgical speciality: general practice, anaesthesiology, occupational medicine, dermatology, Internal medicine, laboratory medicine, microbiology, neurology, nuclear medicine, pathology, paediatrics, physical and rehabilitative medicine, psychiatry, psychosomatics, child and adolescent psychiatry, radiology, radiation therapy, geriatrics, human genetics, palliative care, pharmacology, environment medicine

^b^HSI = Heaviness of Smoking Index (≥2 scores: moderate to high dependence)

^c^Other: pipe, cigar, electro cigarette

^d^AUDIT-C test: (≥5 scores: hazardous alcohol consumption)

^e^One unit equals: 350 ml beer / 150 ml wine/champagne / 40 ml spirits

Response numbers in the category “substance abuse” were too little to allow for valid conclusions. Only five physicians reported regularly taking benzodiazepines, seven regularly consumed Z-drugs (e.g. Zolpidem, Zopiclon and Zaleplon), two opiates and / or opioids, three ecstasy, cocaine and / or amphetamines, and 18 physicians regularly consumed cannabinoids.

### Influencing factors

Comparing the group of people with potentially hazardous alcohol consumption with the group of those who did not consume alcohol or whose consumption was rather harmless in regard to certain risk factors, it is noteworthy that significantly more men than women (32 to 13%) showed a hazardous drinking behaviour (Table 3). Childless persons were significantly more likely (26%) to drink more than parents (19%). Alcohol consumption by physicians working less than 50 h a week was less hazardous (83 to 74%). On the other hand, the age as well as the position and the type of institution did not reveal any significant differences.

**Table 3:** Hazardous alcohol consumption: potential influencing factors (absolute numbers/frequency in %)

Potential influencing factors	Harmless/no alcohol consumption (*n* = 708)	Hazardous alcohol consumption (*n* = 212)	All *n* = 920	*P*-Value
Age				
≤ 35a	251/74	89/26	340	0.089
> 35a	457/79	123/21	580	
Gender				
Female	365/88	52/13	417	< 0,001
Male	343/68	160/32	503	
Maritial status				
Unmarried	215/72	82/28	297	0.075
Married	464/79	122/21	586	
Divorced/widowed	29/78	8/22	37	
Children (living in the household)				
Yes	340/81	82/19	422	0.018
No	368/74	130/26	498	
Position/educational level				
Resident	250/74	88/26	338	0.170
Specialist	199/81	48/19	247	
Physician in a leading position	259/77	76/23	335	
Establishment				
University hospital	284/76	92/26	376	0.657
For-profit/public/non-profit hospital	314/78	87/22	401	
Physician in private practice	110/77	33/23	143	
Working hours per week				
50 h or more	418/74	150/26	568	0.002
Less than 50 h	290/83	62/18	352	

### Consultation behaviour

**Fig. 1:**
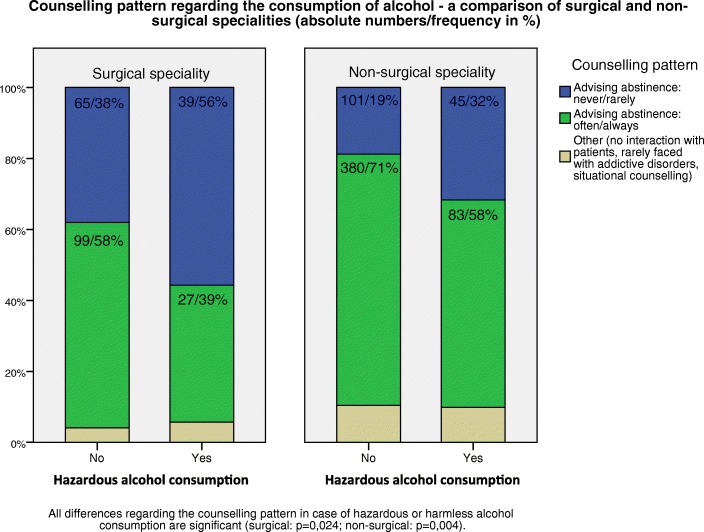
Counselling pattern regarding the consumption of alcohol-a comparison of surgical and non-surgical specialities (absolute numbers/frequency in %)

Doctors who were prone to hazardous alcohol consumption also differed in their counselling pattern (Fig. 1): Regardless of field of study, they rarely or even never advised their patients to abstain from narcotic drugs (surgical: 56%, non-surgical: 32%), compared to doctors without hazardous alcohol consumption.

### CAGE questionnaire

**Fig. 2:**
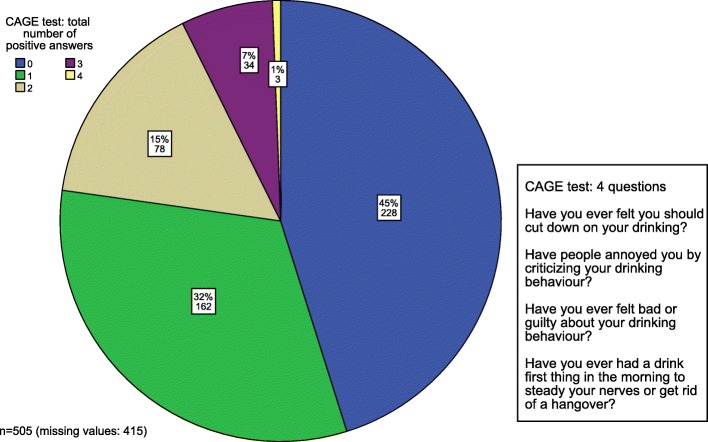
Voluntary CAGE test (frequency in %/absulote numbers)

The CAGE questionnaire was voluntary (Fig. 2). Nevertheless, just over 50% of all participants answered the questions. Since two or more positive answers indicate a positive test, 115 of the responding physicians (12.5% ​​of the total, *n* = 920) would be recommended a further consultation on their alcohol consumption.

**Fig. 3:**
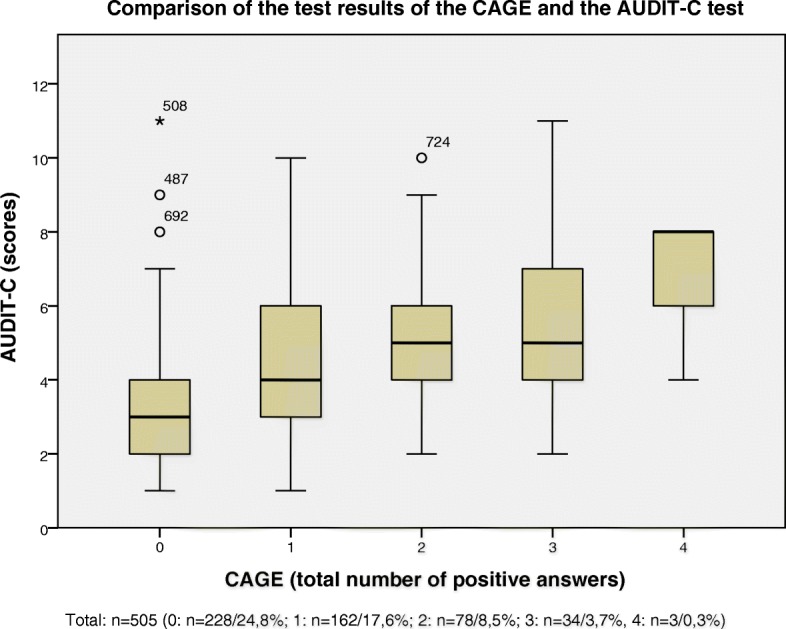
Comparison of the test results of the CAGE and the AUDIT-C test

Figure 3 indicates a positive correlation of the results of the two tests (AUDIT-C; CAGE questionnaire) to evaluate hazardous alcohol consumption. 75% of the physicians, who gave two positive answers, scored 4 or more points in the AUDIT-C test - likewise in the group of physicians who gave three positive answers in the CAGE questionnaire.

### Self-assessment

**Fig. 4:**
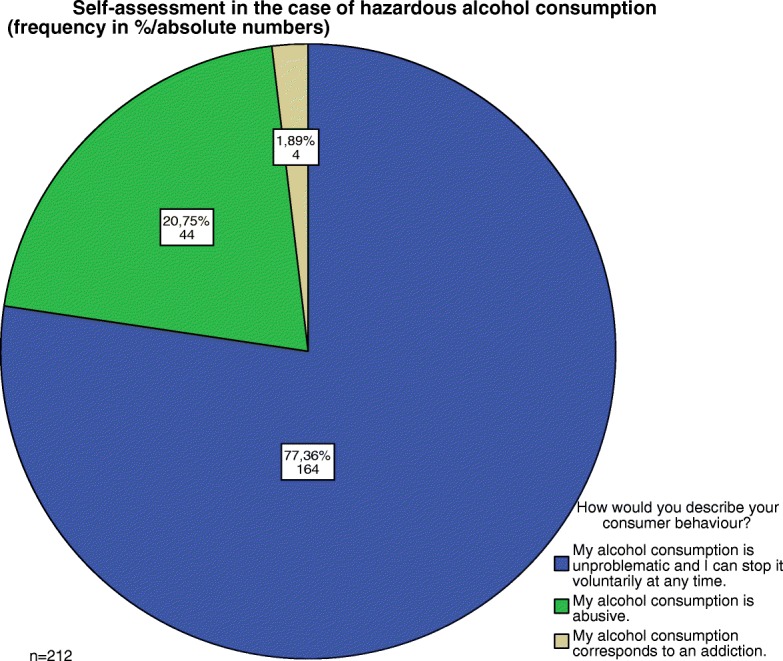
Self-assessment in the case of hazardous alcohol consumption (frequency in %/absolute numbers)

Four people with a positive AUDIT-C test described their alcohol use as a dependency and another 44 as abusive (Fig. 4).

### Regression analysis

To form multivariate logistic regression models, all survey participants were divided into surgical and non-surgical disciplines with regard to their specialization (classification see Table 2). The significant differences in the discipline from Table 2 (“dangerous alcohol consumption”, “risky behaviour”, “prevention investigations”) were further examined with the help of logistic regression. It was adjusted to “age”, “gender”, “children in the household”, “working time”, “subject area”, “position”, “institution”, “burdens”. These were converted into dichotomous variables. Tables 4, 5 and 6 show only the relevant estimators.

**Table 4:** Multivariate logistic regression models^a^: hazardous alcohol consumption

Variables	Odds ratio	95% confidence interval	*P*-value
Hazardous alcohol consumption (AUDIT C test ≥5 points) - all physicians (*n* = 920)			
Female gender	0.27	0,19–0,39	< 0,001
No children in the household	1.67	1,17–2,37	0.004
Female physicians (*n* = 417)			
No children in the household	2.16	1,01–4,62	0.047
Surgical speciality	2.00	0,97–4,12	0.062
Residents^b^	3.10	1,02–9,40	0.046
Specialists^b^	2.61	0,92–7,43	0.071
Male physicians (*n* = 503)			
No children in the household	1.62	1,08 - 2,43	0.019
Surgical speciality	1.27	0,83 - 1,93	0.270
Residents^b^	1.19	0,62 - 2,27	0.605
Specialists^b^	0.66	0,38 - 1,15	0.141

^a^Depending on examined exposure adjusted to: Gender, age, children in the household, medical specialty, position/level of education, establishment, stresses and strains, working hours per week

^b^Compared to physicians in a leading position

**Table 5:** multivariate logistic regression models^a^: risky behaviour

Variables	Odds ratio	95% confidence interval	*P*-value
Risky behaviour - all physicians (*n* = 920)			
Female gender	0.46	0,34–0,63	< 0,001
Working hours per week (> 50 h)	1.56	1,12–2,18	0.009
No children in the household	1.45	1,05–2,00	0.023
Surgical speciality	2.03	1,47–2,81	< 0,001
Female physicians (*n* = 417)			
Working hours per week (> 50 h)	1.69	0,99–2,88	0.055
No children in the household	1.17	0,65–2,08	0.604
Surgical speciality	2.09	1,18–3,69	0.011
Male physicians (*n* = 503)			
Working hours per week (> 50 h)	1.53	0,99–2,38	0.057
No children in the household	1.61	1,08–2,38	0.018
Surgical speciality	2.00	1,34–3,00	0.001

^a^Depending on examined exposure adjusted to: Gender, age, children in the household, medical specialty, position/level of education, establishment, stresses and strains, working hours per week

**Table 6:** Multivariate logistic regression models: preventive check-ups (rare utilization)

Variables	Odds ratio	95% confidence interval	*P*-value
Preventive check-ups - all physicians (*n* = 920)			
Female gender	0.34	0,24–0,48	< 0,001
Surgical speciality	1.57	1,05 - 2,35	0.029
Age < 35a	0.68	0,42 - 1,10	0.117
Female physicians (*n* = 417)			
Surgical speciality	1.22	0,71 - 2,09	0.482
Age < 35a	0.80	0,43 - 1,51	0.490
Male physicians (*n* = 503)			
Surgical speciality	2.15	1,13 - 4,09	0.020
Age < 35a	0.49	0,22 - 1,10	0.083

*Depending on examined exposure adjusted to: Gender, age, children in the household, medical specialty, position/level of education, establishment, stresses and strains, working hours per week

In the overall collective, female gender was a significant protective factor against hazardous alcohol consumption (OR = 0.27) (Table 4). However, childless female doctors represented a risk group that was twice as likely to drink alcohol as their female colleagues (OR = 2.16). Female residents even showed three times higher odds compared to their female colleagues in senior positions (OR = 3.10), but there was a large confidence interval of 1.02–9.40, indicating a too small collective. In the case of male physicians, childless physicians had a significantly increased risk of hazardous alcohol consumption (OR = 1.62).

Table 5 shows that surgeons were twice as likely to behave riskily as their non-surgical counterparts (OR = 2.09 for “female doctors” and 2.00 for “doctors”). Childlessness was associated with risky behaviours among male physicians (OR = 1.61). Similarly, women were significantly less likely to behave riskily than men (OR = 0.46) and physicians who work more than 50 h a week were significantly more likely than their less-active counterparts (OR = 1.56).

Table 6 shows that female doctors attended a significantly higher number of check-ups than male doctors (OR = 0.34). Surgical specialists were less likely to take precautionary measures than their non-surgical counterparts (OR = 1.57). Being a surgeon, especially in males, was clearly associated with the outcome “rare use of check-ups” (OR = 2.15).

## Discussion

### Sample and approach

In order to lose as few participants as possible and to maintain anonymity in the context of the topic, it was decided not to collect confirmations of the internal forwarding by the hospital management and to calculate a response rate from this. In particular, international contact attempts (Great Britain and the USA) proved to be difficult, as there were considerable concerns regarding the confidentiality of data or traceability.

More younger physicians took part in the survey, as they may be more Internet affine than their older colleagues [37]. Furthermore, it is also to be assumed that very sick or stressed doctors do not participate in such a survey. All the more surprising is the high number of senior physicians, as they are exposed to a slightly higher number of weekly working hours than medical residents [38].

Literature suggests that distortions due to the effect of social desirability or false responses play a less relevant role in online surveys, the reason for this being that the Internet provides respondents with an anonymous environment [39, 40]. However, it can be assumed that especially physicians with intentionally risky substance use or already diagnosed dependence - in particular with regard to the medical habitus - for reasons of shame, concern about confidentiality of the data and thus labour law consequences do not participate in such online surveys. It may therefore be assumed that there are a certain number of unreported cases, so that the actual prevalence of risky drug use or risk behaviour may be higher than shown in the present work.

For the AUDIT-C, a cut-off score of 5 points was used for men and women, as it could achieve a higher specificity compared with 3 points (0.42 vs. 0.83) [36]. Admittedly Rumpf et al. found a higher sensitivity for at-risk drinking throughout the AUDIT compared to the AUDIT-C, however, specificity of the latter was higher. This was crucial to avoid overestimating the problem. Moreover, it was not expedient to carry out a screening as in the clinical setting, but to minimize the number of false-positive results so as to better map and reflect the reality. Correction of the specificity results was not performed, therefore the results should be interpreted with caution - a survey among Salzburg physicians showed a corrected value of 7% as a prevalence for high-risk alcohol consumption, uncorrected reached a positive value in the AUDIT-C of 27.4% [41].

Since the CAGE questionnaire had to be completed on a voluntary basis in order not to lose participants through personal questions, only just under half of all participants could be evaluated.

As a whole the questionnaire is not validated and thus the results have to be interpreted very carefully. Partially, validated questions were used, though standardised questionnaires to examine physicians‘ health better lack. This study merely might provide hints on existing difficulties and constitute a first impression of the current situation in Germany.

### Investigated variables

With regard to nicotine consumption, Voigt et al. found the same value for the number of non-smokers in 2009 [18]. However, with regard to smoking behaviour, they only determined the number of cigarettes smoked per day. The Heaviness of Smoking Index - a section of the Fagerström test for nicotine addiction - also makes it possible to distinguish the severity of nicotine dependence [42].

With regard to ethanol, male physicians generally seem to drink more than female doctors, but women in the present survey are significantly more influenced by professional factors such as position and discipline in their drinking behaviour. In contrast to Rosta et al. we could not identify affiliation to surgical disciplines as an independent risk factor for hazardous alcohol consumption for men, whereas for women - if there would be a higher number of surgeons - a high risk of surgical subjects could be identified with high probability - the wide confidence interval indicates a low number of female surgeons in this study (*p*-value 0.062, OR = 2.00, CI: 0.97–4.12) [13, 43]. This corresponds to findings for female surgeons in Norway [43].

Assistants also seem to have a high odd (*p*-value 0.046, OR = 3.10, CI: 1.02–9.40). This raises the question of whether women are less able to compensate for the burden of working in operational subjects than men.

Finally, we were able to confirm the lack of influence of age on hazardous alcohol consumption as described by Rosta et al. [13].

One risk group still consists of childless physicians: they are more prone to risky behaviour and hazardous alcohol consumption - this could be due to the fact that childless doctors have no obligation to be role models for anyone, have no responsibility in educational and entertainment aspects, and possibly more time for hazardous ventures.

While senior and chief physicians with children in literature have an increased risk of burnout, childlessness and long weekly working hours may predispose to more frequent risky behaviour [44]. Surgical professionals and physicians who work more than 50 h per week seem more risk-averse - a coping mechanism may be responsible.

A surgical discipline leads to less frequent use of preventive examinations in men. Men are generally less likely to seek medical check-ups and tend to be more careless about their own health [45, 46]. Surgeons may argue more than others to have to meet a particular personality profile required by the patient and supervisor: great self-esteem, high resilience, strong stamina. Illness could be interpreted as a sign of weakness and preventive examinations are therefore avoided.

In conclusion, however, risky behaviour, as well as the use of preventive screening and high-risk alcohol consumption, seems to be independent of whether or not respondents suffered from stress at the time of the survey.

For comparison no current data is available regarding the prevalence of hazardous alcohol consumption in certain occupation groups apart from physicians in Germany. Documented data on problems caused by alcohol in the professional environment can provide information on exposed occupation groups: Especially affected seem to be people who are working in the gastronomy. Also the category “delivery of other economic services” which includes temporary work is affected above average [47]. Regarding the total population the following data should be mentioned: the prevalence of alcohol-related disorders by DSM-IV in adults between 18 and 64 years in Germany in 2013 evaluated by Pabst et al. for abuse and dependency in total 6,5%, in men 9,5% and in women 3,5% [48]. A study by the Robert-Koch-Institute from 2008 till 2011 by the means of the AUDIT-C test resulted in a risky consumption of alcohol for 41,6% for the men and 25,6% for the women [49].

## Conclusion

Risk groups and factors influencing health-endangering behaviour may further be investigated in future studies and standardised questionnaires for the examination of physicians‘health have to be developed. The number of women in the medical profession will continue to increase and so will the need for female doctors in surgical professions [50, 51]. These require a high level of resilience and coping strategies as stress and strain levels increase. There is an urgent need to conduct further studies on the risk for female surgeons for hazardous alcohol consumption.

In Germany aid offers are readily available. Specialist clinics, such as the Oberbergkliniken, provide an anonymous environment and offer structured treatment options [52]. In cooperation with the regional medical associations, there is an intervention program based on the principle “help instead of punishment”, which addresses the special circumstances of the medical profession: It guarantees anonymity, regulates practice representation and reimbursement of costs, and offers close outpatient follow-up care [4, 53].

However, the primary prevention should be extended significantly, as many doctors are not familiar with the offers of help and treatment opportunities and, among other things, do not get help for fear of labour-law consequences. To address this, one could also train chief physicians better in the handling and recognition of addiction problems. One could also teach doctors how to interact with and help potentially affected colleagues. There should already be contact persons in the immediate vicinity of the clinic. For example, with the help of an occupational medical burnout screening to identify particularly stressed physicians who have few coping strategies, risk groups could be identified at an early stage.

### Key messages

- Nearly one in four physicians consumed alcohol at hazardous levels.
- Female assistants had a three times higher risk of hazardous alcohol use compared to women in senior positions.
- Surgical professionals tended towards risky health habits twice as often compared to their colleagues in non-surgical disciplines.
- There is a need to improve primary prevention.
- The aim should be the identification of the most vulnerable groups followed by early intervention.
